# Gems from Many Authors

**Published:** 1882-12

**Authors:** 


### GEMS FROM MANY AUTHORS.

The dew of compassion is a tear.—*Byron.*

Genius is eternal patience.—*Michael Angelo.*

A woman's fitness comes by fits.—*Shakespeare.*

Heaven gives its favorites early death.—*Byron.*

Women, like Princes, find few real friends.—*Lord Lyttleton.*

The society of women is the element of good manners.—*Goethe.*

You will find poetry nowhere unless you have some with you.—*Joubert.*

None but the guilty can be long and completely miserable.—*Goldsmith.*

A curious fact—Satan deprived Job of everything except his wife. —*Anon.*

Beauty is the first present nature gives to woman, and the first it takes away.—*Moore.*

Bad men hate sin through fear of punishment. Good men hate sin through love of virtue.—*Juvenal.*

Thou wilt be great only in proportion as thou are gentle and courageous to subdue thy passions.—*Fenelon.*

One must feel intellectually secure before he can begin to dress shabbily ; no one but a genius or a great scholar dares to be dirty. —*Irving.*

The right of commanding is no longer an advantage transmitted by nature like an inheritance ; it is the fruit of labors, the price of courage.— *Voltaire.*

It is better to meet danger than to wait for it. He that is on a lee shore, and foresees a hurricane, stands out to sea, and encounters a storm to avoid shipwreck.— *Colton.*

Use dispatch. Remember the world only took six days to create. Ask me for whatever you please except time ; that is the only thing which is beyond my power.—*Napoleon.*

> My cares are blessed thistles unto me,
> Which wholesome are, although they bitter be ;
> And, though their leaves with pricks are overgrown
> (Which pain me), yet their flowers are full of down.—*George Wither.*

The good things which belong to prosperity are to be wished, but the good things which belong to adversity are to be admired. The virtue of prosperity is temperance, the virtue of adversity fortitude, which in morals is the more heroic virtue.—*Bacon.*

The church of to-day needs to be a believing church, a witnessing church, a working church, a church whose individual members shall exert a fashioning influence on the communities in which they live, doing what they can to make men think aright and act aright toward God and man.—*Zion's Herald.*

